# Developing Correlates of Protection for Vaccines Is Needed More than Ever—Influenza, COVID-19 and RSV Infection

**DOI:** 10.3390/v16111671

**Published:** 2024-10-25

**Authors:** Zoltan Vajo, Csaba Laszlofy

**Affiliations:** 1 Department of Family Medicine, Semmelweis University Medical School, 1085 Budapest, Hungary; 2 Department of Prosthodontics, Faculty of Dentistry, University of Szeged, 6720 Szeged, Hungary; dr.csaba.laszlofy@sanoral.com

**Keywords:** Influenza, COVID-19, RSV, antibody, immunity

## Abstract

One of the greatest success stories of modern medicine is the prevention of infectious diseases by vaccination, most notably against smallpox and poliomyelitis. However, recent events, such as the 2009–2010 swine flu and the 2020 COVID-19 pandemics, as well as the continued emergence of highly pathogenic avian influenza viruses highlighted the fact that we still need to develop new vaccines, and perhaps we should be proactive, rather than reacting to epidemics and pandemics. However, the development of tools for evaluating novel vaccines has not been able to keep up with the rate of vaccine production. Humoral and cellular immune responses to vaccination have both been suggested to be important in preventing infections or ameliorating their consequences, although there is uncertainty regarding their exact roles and importance. This, together with the rapid development of new vaccines, means that the need for developing immunogenicity parameters, and even more importantly, reliable correlates of protection, is more important than ever.

## 1. Introduction

### 1.1. Past Achievements

One of the greatest victories achieved by the medical science is the one against infectious diseases. In the not-too-distant past, diseases caused by microbes represented the top cause of mortality. For centuries, infectious diseases killed tens of millions of people, causing multiple epidemics and pandemics. An outbreak of the bubonic plague in London in 1665 killed nearly 25% of London’s population, while the Spanish flu in 1918 caused more deaths in a few months than World War I itself claimed in four years [1]. Developing safe and effective antimicrobials changed this picture dramatically, and the goal shifted towards preventing infectious diseases when possible. One of the most effective ways of achieving this is vaccination, which dates all the way back to 1796, as far as Western medicine is considered, but perhaps much earlier in some other societies [2]. Thanks to vaccines, a virus that caused a devastating disease for 3000 years, smallpox, has been eradicated, and the effects of many more have been largely reduced [3].

### 1.2. Future Challenges

Despite the above, recent events, such as the 2009–2010 swine flu and the 2020 COVID-19 pandemics, as well as the continued emergence of highly pathogenic avian influenza viruses, highlighted the fact that we still need to develop new vaccines, and perhaps we should be proactive, rather than reacting to pandemics. Due to that realization, as well as the discoveries of novel vaccine platforms, most notably nucleic acid-based vaccines, the development of new vaccines has never been more rapid than it has been in the past decade. We are currently developing vaccines against microbes that have not caused a pandemic (yet), such as chikungunya, zika or highly pathogenic avian influenza (HPAI) viruses [4,5]. However, the development of tools for evaluating novel vaccines has not been able to keep up with the rate of vaccine production, as highlighted by the rapid approval and deployment of COVID-19 vaccines. Humoral and cellular immune responses have both been suggested to be important in preventing infections and/or ameliorating their consequences, although their exact roles and importance are uncertain [6]. This means that the need for developing novel immunogenicity parameters, and even more importantly, reliable and specific correlates of protection, is more important than ever.

## 2. Influenza

### 2.1. Trivalent and Quadrivalent Inactivated and Recombinant Influenza Vaccines

The majority of influenza vaccines are trivalent or quadrivalent, inactivated or recombinant vaccines. These can be further categorized as egg-based or cell culture-based, standard vaccines or high-dose vaccines, adjuvanted vs. non-adjuvanted and split virion, protein subunit or, in some countries, whole virion, influenza vaccines [7,8]. For decades, we thought that influenza was perhaps the best understood viral infection, as far as natural and vaccine-generated immunity is concerned. Thus, well-described immunogenicity criteria have been set by the European Medicines Agency, as well as by the US Food and Drug Administration (FDA), for the licensing of newly developed vaccines, and even for the yearly release of updated seasonal vaccines [9,10]. This mainly involves the hemagglutination inhibition (HAI) assay, which indirectly measures antibodies against one of the main viral proteins, hemagglutinin. Based on human challenge studies from half a century ago, it was generally accepted that a HAI titer of 1:40 correlates with approximately 50% protection against the disease in healthy adults [11]. Therefore, the European and American regulatory agencies established the following three licensing criteria for seasonal influenza vaccines: 40% seroconversion (meaning either a negative prevaccination serum and a postvaccination serum titer of >1:40, or an at least a fourfold increase in the titer postvaccination), 70% seroprotection (the percentage of patients showing at least a 1:40 HAI titer after vaccination) and a post/prevaccination Geometric Mean Titer ratio increase of 2.5 [9,10] (Table 1). This was slightly modified for elderly patients, lowering the licensing immunogenicity criteria for vaccines to 30% seroconversion and 60% seroprotection, although it was shown that in elderly patients, 60% of infected individuals had titers ≥1:40, and 31% had titers even as high as ≥1:640. This clearly shows that the regular cutoff HAI titer of 1:40 cannot be applied as an approximate correlate of protection in the elderly population [12]. Moreover, no separate licensing criteria existed for pediatric populations, despite some evidence that the generally accepted 1:40 HAI titer might not accurately represent 50% protection for them either [13]. Furthermore, even in healthy adults, a meta-analysis showed a strong variation in the HAI titers required to obtain a 50% protection from influenza [14].

Based on the above, even in the case of one of the most studied vaccines, influenza, the need to develop more accurate immunogenicity markers to correlate well with real-world protection still exists. These might involve virus neutralization, neuraminidase inhibition, single radial hemolysis and various cellular immunity assays [15]. Among these, so far, only single radial hemolysis has been established as a potentially meaningful correlate of protection, as 25 mm^2^ area of hemolysis is thought to be equivalent to an HAI titer of 1:40 and thus correlates with approximately 50% vaccine efficacy [16].

Another test currently under consideration to be included into licensing criteria is microneutralization, especially since some hemagglutinins, notably influenza A H5 and some other highly pathogenic avian influenza strains, are known not to react optimally with the conventionally used chicken erythrocytes during HAI [17].

Based on the experience gained from studies with seasonal influenza, the FDA also developed a guidance for licensing and stockpiling pandemic influenza vaccines [18]. Nonetheless, these are based on the criteria described above. In addition, the WHO developed its own guidance and action plan for stockpiling and deploying pandemic influenza vaccines [19].

### 2.2. Live Attenuated Influenza Vaccines (LAIVs)

The use of LAIVs in the form of nasal sprays, such as the FDA-approved Flumist, represents an additional challenge in terms of finding a correlation between immunogenicity and real-world protection [8]. LAIV preparations have been shown to induce poor serum immune responses, measured by HAI or virus neutralization assays, although they were clinically found to be highly efficacious against influenza in children, as well as in young and middle-aged adults [20]. Besides the conventional HAI and single radial hemolysis, correlates of immune protection for this particular vaccine type should also include secretory immunoglobulin A measurements, although these are much less studied, and no clear cutoff values for protection have been established.

In the context of live vaccines, it should be considered that several different vaccines can have non-specific effects on immune responses, morbidity and mortality. The bacillus Calmette–Guérin (BCG), diphtheria–tetanus–whole cell pertussis (DTPw) and measles vaccines have all been suggested to have effects on mortality that were more than what could be expected through their effects on the diseases they prevent. The mechanisms behind the non-specific effect of vaccines, also known as heterologous effects, are incompletely understood. Nonetheless, they are most likely related to a combination of different effects on the innate and adaptive immune responses, heterologous T-cell responses and influences on responses to other subsequent immunizations [21].

Based on the above, even though influenza vaccines have been used for seven decades, the mechanism and correlates of the protection they provide still need further studies. This could include changing the focus from HAI, which seems to be an unreliable correlate of protection, especially in the elderly population, to other assays of humoral immunity, as well as the relatively less studied cellular immunity. Since vaccine production has to begin at least several months prior to the beginning of the influenza season to generate an appropriate number of doses, the actual circulating virus may differ from the vaccine strain. In such cases, a decreased level of protection is expected, and performing HAI testing against the vaccine virus strain might even be less reliably correlated with real-world protection [22]. Furthermore, in efforts to develop universal influenza vaccines, antigens other than the relatively well-studied hemagglutinin are being investigated. Such alternative antigens include the matrix protein M1, the membrane protein M2, as well as the nucleoprotein (NP). Novel vaccines based on such alternative antigens will require new correlates of protection [23].

## 3. COVID-19

The appearance of SARS-CoV-2 and the resulting pandemic triggered vaccine development at rates that had never been seen before. Multiple manufacturers attempted to license vaccines worldwide, even though they used novel vaccine platforms. This presented an unprecedented challenge to the licensing authorities. One mitigating factor was that during the development of SARS-CoV-2 vaccines, the disease, COVID-19, was already highly prevalent in the general population. Thus, enrolling subjects in clinical trials and gaining sufficient statistical power was easier than in cases of some other diseases, where the infection was much less common.

The spike protein of the virus, or protein S, was selected as an important target for vaccine production, including mRNA, adenoviral vector DNA and recombinant protein vaccines, and antiviral medication development. Its subunit, the receptor-binding domain (RBD) is a key determinant of viral infectivity, and hence, it is considered to be the main antigenic component that is responsible for inducing a host humoral immune response. As a consequence, the developers of laboratory tests focused mainly on producing assays for anti Spike protein antibodies, mainly IgG, since most vaccines, i.e., the mRNA (by Pfizer/BioNtech and Moderna), DNA vector (most notably by Janssen and Astra Zeneca), and recombinant protein vaccines, such as the product by Novavax, all induced antibodies against that single immunological target. Thus, developing high levels of antibodies against the receptor-binding domain of the spike protein of the virus was considered evidence of protection, although no widely accepted cutoff titers were established. Initially, the concept was simple: the higher the concentration of the anti-spike protein antibodies are, usually expressed in artificial units (AU) per milliliter, the more likely it is that the individual is protected, either by vaccination or natural infection. However, it quickly became evident that there was more to the story, as rapidly declining levels of such antibodies do not necessarily mean fading or ceasing protection [24]. In addition, while it appeared that although most vaccines induce comparable rises in the concentrations of anti-spike protein antibodies, the level of protection they provide might be different. In addition, virus neutralizing assays have also been developed and utilized in clinical trials, albeit with the same limitations as discussed above.

Some trials suggested cutoff values in the case of humoral immunity parameters for protection against SARS-CoV-2 [25,26]. In a widely cited study, Khoury et. al. proposed that a 50% protective neutralization level equivalent to 20% of the mean titer in the convalescent subjects equates to a measured in vitro neutralization titer of between 1:10 and 1:30 in most clinical trials (although in some cases, it reached up to 1:200). It is estimated that this corresponds to approximately 54 international units (IU)/mL (95% CI 30–96 IU/mL) [26]. Nonetheless, these are not part of the licensing process for COVID-19 vaccines at this time.

Establishing antibody titers as a correlate of protection and defining a protective titer would be extremely important for public health considerations and patient care. A reliable correlate of protection and a protective threshold would also allow for developing new SARS-CoV-2 vaccines based on smaller immunogenicity-based phase 3 trials instead of large and expensive field vaccine efficacy trials, which are becoming increasingly difficult to perform.

In addition to the above vaccines immunizing against a sole antigen, Protein S, several manufacturers, notably in China (Sinopharm and Sinovac), India (Bharat Biotech) and Europe (Valneva, France), produced vaccines with more conventional methods, containing the entire virion in inactivated vaccines, and thus providing multiple viral antigens. Therefore, in those cases, testing for antibodies against the nucleocapside protein of SARS-CoV-2 was also utilized [27]. Unlike with influenza, the protective antibody titer for SARS-CoV-2 is still unknown, although non-human primate studies suggest that it is likely to be relatively low [28]. Nonetheless, growing evidence has suggested, again, similarly to influenza, the important role of cellular immunity, especially CD8+ T cells, in developing protection against COVID-19 [6], although it is less likely to be induced by viral protein-based vaccines [29]. Cellular immunity against SARS-CoV-2 can be assessed by multiple methods, such as measuring IFNγ production by ELISPOT or measuring intracellular cytokines or cytotoxic granules by flow cytometry. However, despite extensive research, no clear cutoff values of protection have been established to date. Establishing such parameters by further studies on humoral and cellular immunity would undoubtedly aid not only in vaccine development and licensing, but also in understanding the complex natural immune reaction to SARS-CoV-2 infection.

## 4. Respiratory Syncytial Virus, RSV Infection

Human respiratory syncytial virus (RSV) is an enveloped, negative-sense, single-stranded RNA virus belonging to the Pneumoviridae family. It is one of the leading causes of lower respiratory tract infections, including pneumonia and severe bronchiolitis in pediatric, elderly and immune-compromised patients. Natural RSV infection usually does not induce lifelong immunity, and re-infections can occur throughout an individual’s lifespan. As of today, there are no immune markers widely accepted as predictive of protection against re-infection with RSV. The durability and precise mechanism of the naturally acquired immunity after RSV infection is not completely known.

Some of the newest vaccines address the large health care burden caused by RSV infections [30], as the most recent vaccine development includes those against RSV. Currently, there are two FDA-approved RSV vaccines, namely Arexvy and Abrysvo, both of which are recombinant, stabilized prefusion F-protein (preF) vaccines, with or without adjuvants, respectively. Hence, they both utilize a single viral antigen. Thus, antibody testing against the preF appears to be the logical approach to assess vaccine immunogenicity. The levels of RSVPreF3-specific immunoglobulin G [IgG] and RSV-A neutralizing antibodies were found to be significantly higher after vaccine administration [31]. However, to date, no clear correlation or cutoff levels for protection have been established for consensus. As of cellular immunity, compared to prevaccination, the geometric mean frequencies of polyfunctional RSVPreF3-specific CD4+ T cells increased after each RSV vaccine dose. This indicates that the RSV vaccines induce a measurable cellular immune reaction; however, the precise role of this response and its correlation to protection have not been established. On the other hand, vaccination with RSVPreF3 vaccines as expected, did not measurably increase CD8+ T-cell responses compared to placebo [31]. Nonetheless, the vaccine was found to provide 94.1% protection against severe RSV-related lower respiratory tract disease in elderly adult patients [32]. This, again, highlights the fact that humoral and cellular vaccine immunogenicity and its relation to efficacy are not completely understood.

Further studies on the immunogenicity of RSV vaccines in preclinical and clinical studies, including animal challenge, and prospective, randomized human trials will, without a doubt, help to not only understand the mechanism behind the protective actions of vaccines but also the immune response to natural infection.

## 5. Conclusions

Studying vaccine immunogenicity and developing surrogate markers for protection after vaccination or infections is of great importance. This will allow for the assessment of sufficient individual immune responses after vaccination, or, as was the case with influenza vaccines in the past, will even simplify and shorten the licensing process. The rate at which we have been developing vaccines against long-existing and newly emerging microbes has been accelerating rapidly. This was further facilitated by the COVID-19 pandemic and the approval of novel vaccine platforms. As further pandemics may approach, such as highly pathogenic avian influenza (HPAI), chikungunya, zika, dengue or other viruses, rapid and effective vaccine development is likely to remain a high public health priority. Thus, developing tools to assess vaccine immunogenicity and finding reliable surrogate markers for real-world protection is more important than ever before. To achieve that goal, head-to-head, randomized controlled clinical trials involving multiple vaccines to assess their real-world protection, along with their immunogenicity, are much needed.

## Figures and Tables

**Table 1:** Previous EMA and current FDA guidelines for the immunogenicity parameters for influenza vaccines in adult and elderly patients.

	Adult (Age: 18–65 Years)	Elderly (Age > 65 Years)
**Immunogenicity criteria:**	≥40%	≥30%
-Percentage of subjects with seroconversion * or significant increase in titers.		
-Percentage of subjects seroprotected **	≥70%	≥60%
-GMT fold rise (post/prevaccination ratio) ***	>2.5	>2.5

* Seroconversion is defined as either a negative prevaccination serum with a postvaccination serum titer of >1:40, or an at least fourfold increase in titer postvaccination. ** Seroprotection is defined as achieving at least a 1:40 titer of hemagglutination inhibition. *** The FDA does not use this parameter.

## Data Availability

No new data were created or analyzed in this study. Data sharing is not applicable to this article.
